# Web-Based Immersive Virtual Patient Simulators: Positive Effect on Clinical Reasoning in Medical Education

**DOI:** 10.2196/jmir.5035

**Published:** 2015-11-17

**Authors:** Robert Kleinert, Nadine Heiermann, Patrick Sven Plum, Roger Wahba, De-Hua Chang, Martin Maus, Seung-Hun Chon, Arnulf H Hoelscher, Dirk Ludger Stippel

**Affiliations:** ^1^Department of General, Visceral, and Cancer SurgeryUniversity of CologneCologneGermany; ^2^Department of RadiologyUniversity of CologneCologneGermany

**Keywords:** medical education, simulation, virtual patients, immersive

## Abstract

**Background:**

Clinical reasoning is based on the declarative and procedural knowledge of workflows in clinical medicine. Educational approaches such as problem-based learning or mannequin simulators support learning of procedural knowledge. Immersive patient simulators (IPSs) go one step further as they allow an illusionary immersion into a synthetic world. Students can freely navigate an avatar through a three-dimensional environment, interact with the virtual surroundings, and treat virtual patients. By playful learning with IPS, medical workflows can be repetitively trained and internalized. As there are only a few university-driven IPS with a profound amount of medical knowledge available, we developed a university-based IPS framework. Our simulator is free to use and combines a high degree of immersion with in-depth medical content. By adding disease-specific content modules, the simulator framework can be expanded depending on the curricular demands. However, these new educational tools compete with the traditional teaching

**Objective:**

It was our aim to develop an educational content module that teaches clinical and therapeutic workflows in surgical oncology. Furthermore, we wanted to examine how the use of this module affects student performance.

**Methods:**

The new module was based on the declarative and procedural learning targets of the official German medical examination regulations. The module was added to our custom-made IPS named ALICE (Artificial Learning Interface for Clinical Education). ALICE was evaluated on 62 third-year students.

**Results:**

Students showed a high degree of motivation when using the simulator as most of them had fun using it. ALICE showed positive impact on clinical reasoning as there was a significant improvement in determining the correct therapy after using the simulator. ALICE positively impacted the rise in declarative knowledge as there was improvement in answering multiple-choice questions before and after simulator use.

**Conclusions:**

ALICE has a positive effect on knowledge gain and raises students’ motivation. It is a suitable tool for supporting clinical education in the blended learning context.

## Introduction

One important part of clinical education is learning and mastering medical workflows, which is the basis for mastering clinical workflows in diagnosis and therapy (clinical reasoning). Nowadays, many workflows are based on standard operation procedures (SOPs) that enable performance uniformity in the clinical daily routine [1]. A key basis of these procedures is the learning of the underlying (declarative) knowledge, which is nowadays often supported by e-learning programs [2]. Mastering SOPs require the ability to transfer declarative knowledge (what to do) into practical or procedural knowledge (how to do it). This is most effective in attending courses, preferably in small groups where students’ preexisting knowledge is on the same level [3]. In current medical curricula, new educational approaches such as problem-based learning, skills lab, and mannequin simulators [4,5] play an increasing role. By repetitive training of standardized clinical settings, procedural knowledge can be internalized, which ensures a certain knowledge level before working with real patients.

The rapid development of computer technology enables implementation of new educational approaches for teaching and internalizing medical workflows in diagnosis and therapy even on home computers. Immersive patient simulators (IPSs) enable a representation of a virtual counterpart in a three-dimensional “game-like” virtual environment where students can freely interact in real time with virtual patients [6]. Playful immersion in the digital environment provides virtual experience as students can face the consequences of different decisions (trial and error) without putting real patients at risk. Repetitive training allows internalization and consolidation of the scripts that are relevant for the necessary procedural performance [7]. Furthermore, Web-based IPS combines immersion with the advantages of distance learning [8].

In a thematic review, we summarized the available immersive virtual patient simulators and found that the use of such simulators in clinical education is still rare. Furthermore, there was no IPS available that combined high technical quality with an in-depth level of medical content and that was freely accessible for all students [9]. Therefore, we developed a university-based IPS prototype called “ALICE” (Artificial Interface for Clinical Education) that is free to use, available to all interested teachers, and that enjoys high student acceptance [10]. However, application of this new educational approach requires knowledge about the impact on knowledge gain as clinical teachers may ask whether using a simulator of this kind can really support clinical education.

We formulated the hypothesis that repetitive training with ALICE consolidates clinical reasoning and has a positive impact on knowledge gain. Therefore, we implemented a teaching module that simulates patients with a complex oncological disease. It was our aim to perform a validation of this novel educational approach and to examine whether the use of ALICE has positive impact on clinical reasoning and is a suitable tool for supporting the clinical teacher.

## Methods

### Immersive Virtual Patient Simulator: ALICE

**Figure 1:**
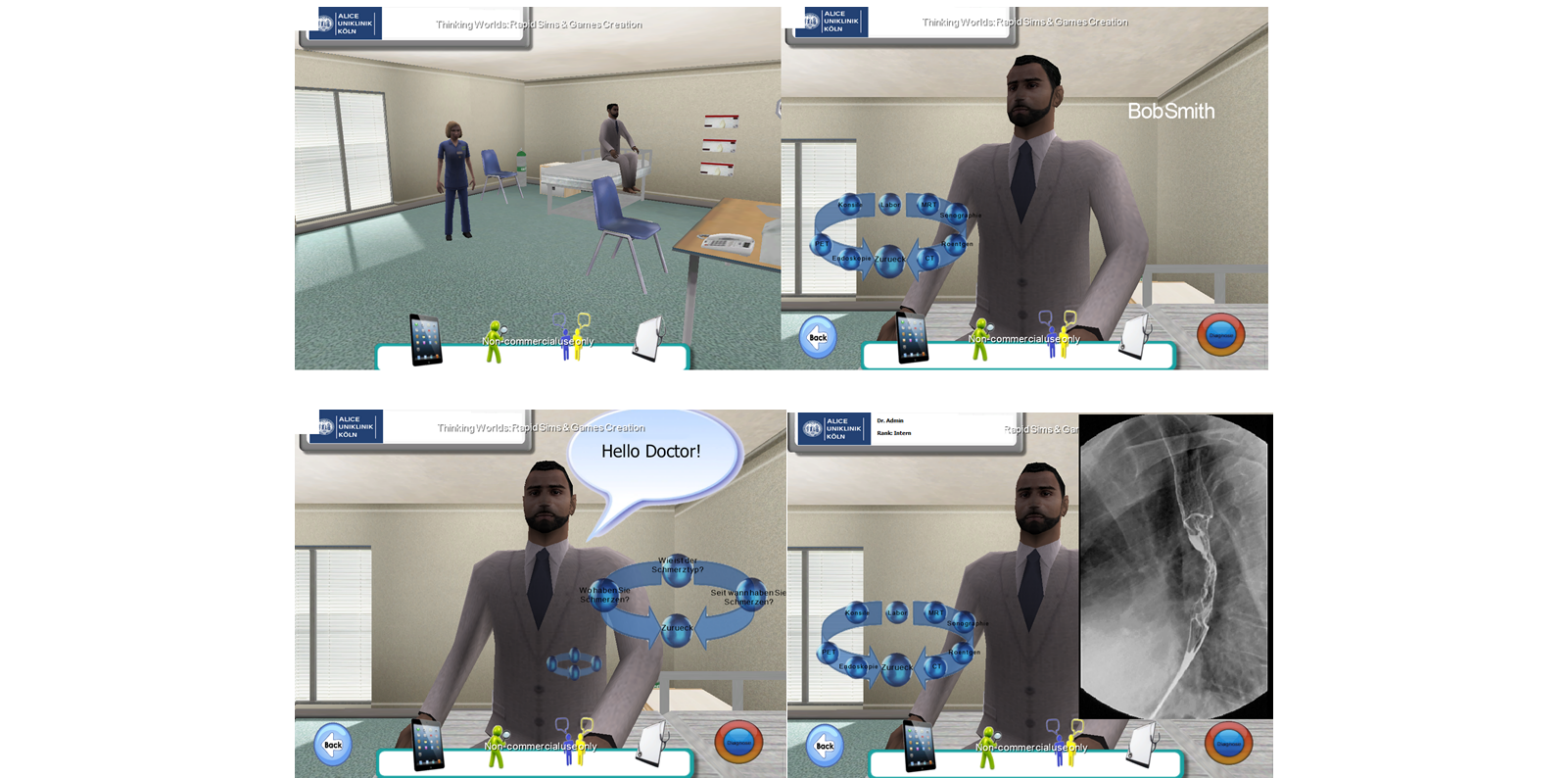
Screenshot of ALICE. Students can freely navigate an avatar through a virtual outpatient clinic (upper left) and freely interact with the virtual patient via a dynamic graphical user interface (GUI) (upper right). Students can communicate with the virtual patient (lower left) via the GUI and choose different medical examinations. Results of these examinations are presented either as image, text or video and remain uncommented at this point (lower right).

**Figure 2:**
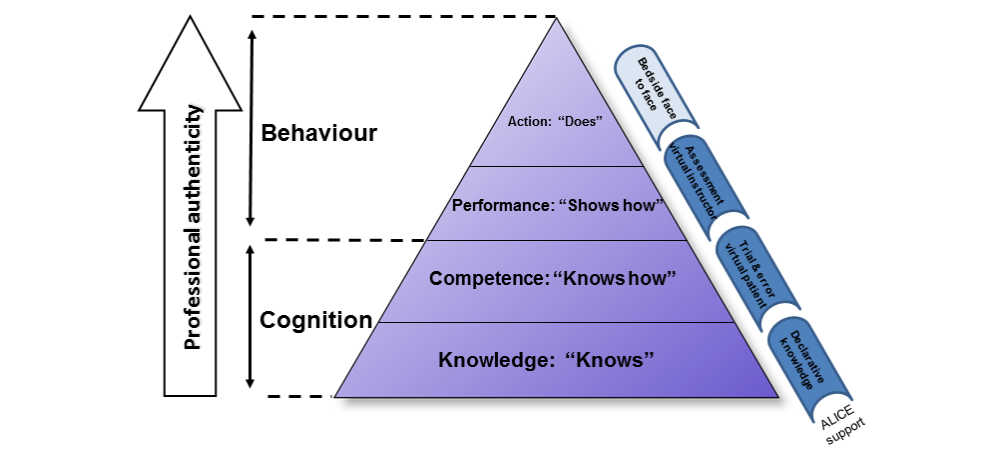
Simulator design is based on the concept of Miller’s pyramid (upper part) and was developed to support clinical education in the first two steps. Modified from Miller 1990 [11].

**Figure 3:**
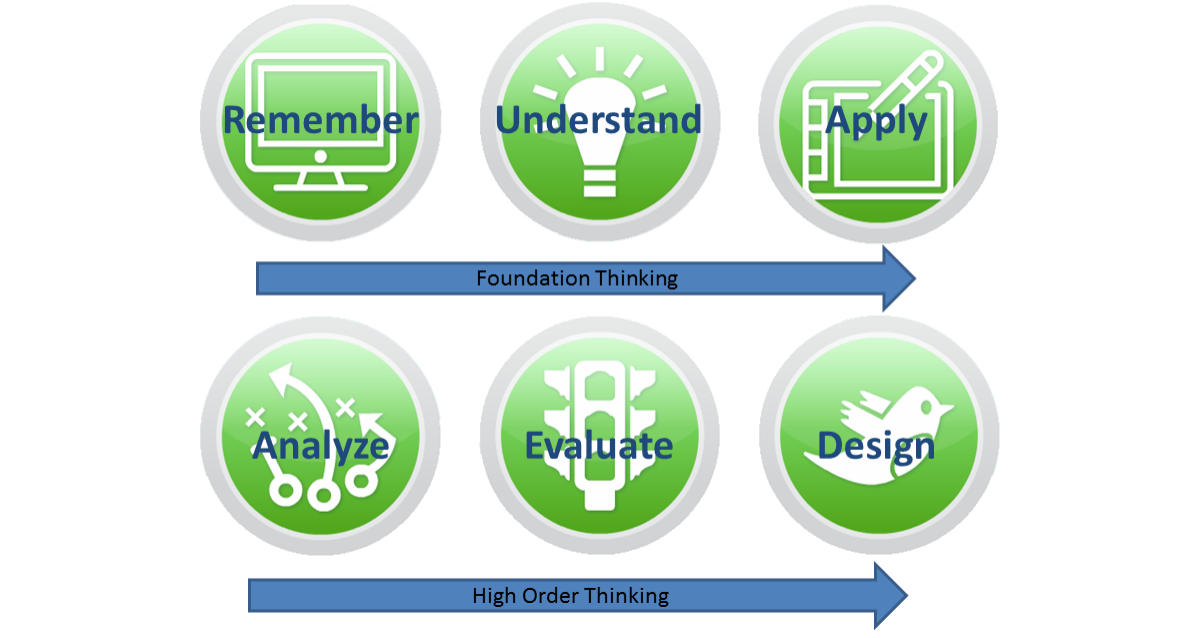
Bloom’s taxonomy of the cognitive domain: ALICE supports learning on the first three steps (Foundation Thinking).

Design and technical realization were previously described by our group [10]. In brief, ALICE is a Web-based IPS that enables the student to navigate through a virtual environment from a first-person perspective similar to a video game (Figure 1). ALICE simulates a small outpatient clinic with a treatment room where the user can interact with nonplayer characters such as nurses, patients, and other doctors (Figure 1). The simulator starts with a short instructional case where the basic controls and functions are explained. The user is able to freely interact with the virtual patient and choose the different options (medical history, examination, diagnostic tests). The student instantly receives the desired result either as a table (eg, laboratory values), image (eg, x-ray, electrocardiogram), or video (eg, computed tomography scans, ultrasound, gastroscopy). When choosing a medically nonindicated examination, the test shows a normal finding. Once the student makes a diagnosis, he or she initiates the necessary treatments and finishes the case. The models of Miller’s pyramid [11] and Bloom’s taxonomy were used as a scaffold for planning and assessing our simulator (Figure 2). Level of competence was assessed on level one (factual test), level two (clinical context-based test), and partly on level three of Miller’s pyramid (performance assessment in simulated patients). ALICE helps students gain and understand knowledge and offers the opportunity to apply this knowledge on virtual patients, which are important steps in Bloom’s taxonomy in the cognitive domain [12] (Figure 3).

### Adding Medical Content

ALICE can easily be expanded by adding content modules that include a specific disease. These teaching modules contain all information about the underlying declarative and procedural knowledge as well as patient characteristics and images. These modules can be added via a special editor. Determination of impact on clinical reasoning required the development of a new simulator module. To support or reject the hypothesis that repetitive simulator use can consolidate clinical reasoning, the following requirements were defined: The simulator should mimic a disease with a standardized workflow in diagnosis and therapy. It is mandatory that there are concurrent therapeutical concepts that depend on specific clinical findings. Moreover, this specific disease should not have been a focus in the previous clinical curriculum, to minimize the influence of already obtained knowledge.

### Study Design

**Figure 4:**
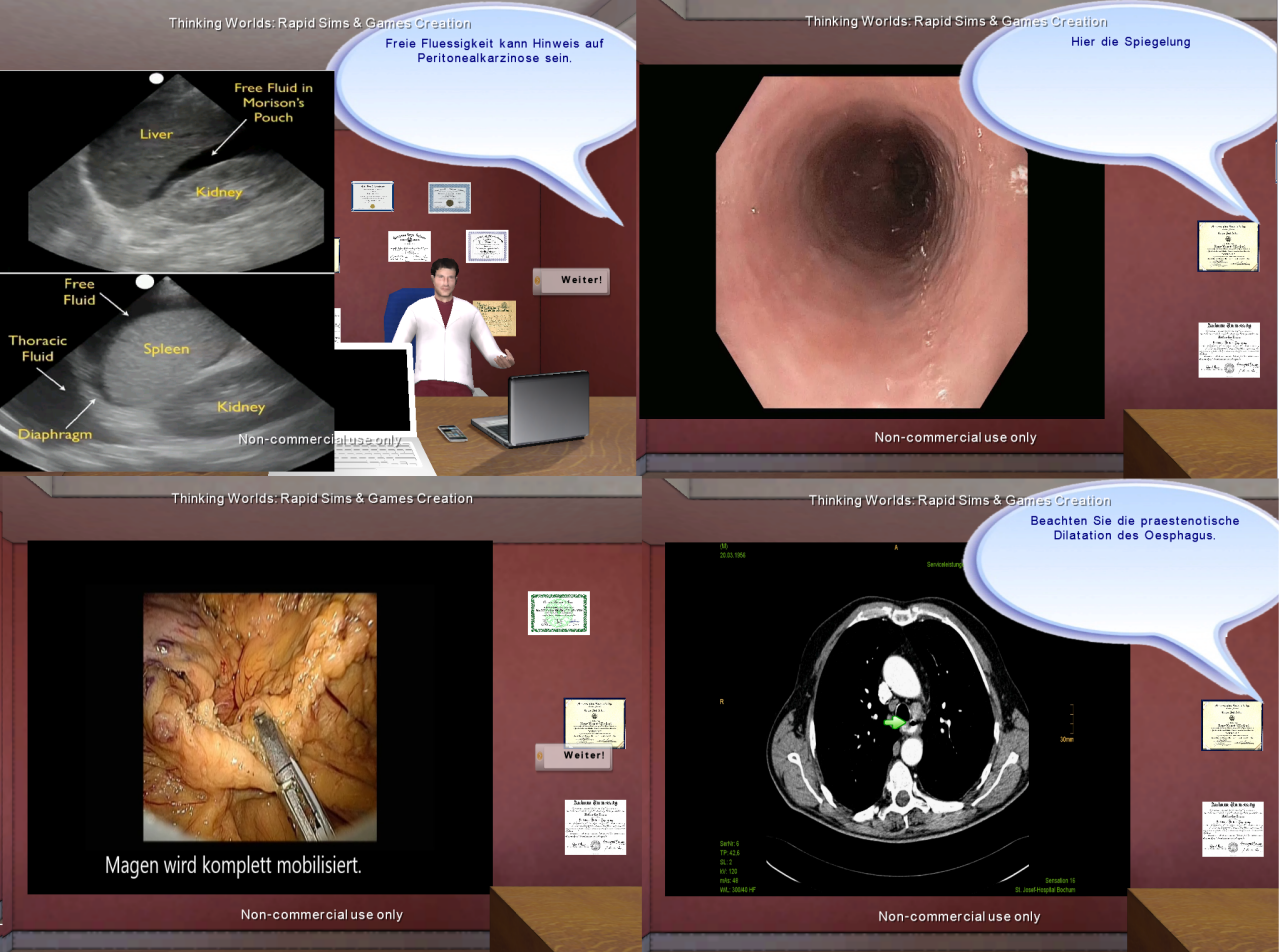
Debriefing scene represents a virtual instructor who explains and summarizes the different findings. Debriefing ends with a video presentation of the specific surgical procedure and a summary of the underlying declarative knowledge.

The module was evaluated in the context of a surgical seminar held at the surgical faculty of the University of Cologne. A total of 62 third-year students were taught in a small group (<5 students), each accompanied by an experienced board-certified surgeon. Each student received the patient files from 2 real patients diagnosed with esophageal cancer. Students were not aware of the diagnosis. Students were asked to summarize the findings in a clinical reasoning summary. This included diagnosis, diagnostic tests, and suggested therapy. In addition, students answered multiple-choice questions taken from the official state examination board to determine the preexisting declarative knowledge about the underlying disease. This presimulator questionnaire was stored for later analysis. After completion of the presimulator questionnaire, students worked with ALICE. Students’ performance was reviewed immediately after each case as procedural review of students’ performance immediately after training is known to be an effective incentive for knowledge gain [13]. A virtual instructor summarizes the performance based on a short comparison of the students’ choices and stored SOPs. Furthermore, the virtual instructor summarizes the underlying declarative knowledge and optimal procedural pattern. The debriefing ends with a video presentation of the corresponding surgical procedure and a summary of the underlying declarative knowledge (Figure 4).

After completing the simulator cases, students had to perform a reassessment of the clinical reasoning of the 2 patients and complete a postsimulator questionnaire that contained the same questions as before. Cases were recapped and summarized after simulator evaluation by the clinical teacher in a small-group, face-to-face learning.

### Evaluation

**Figure 5:**
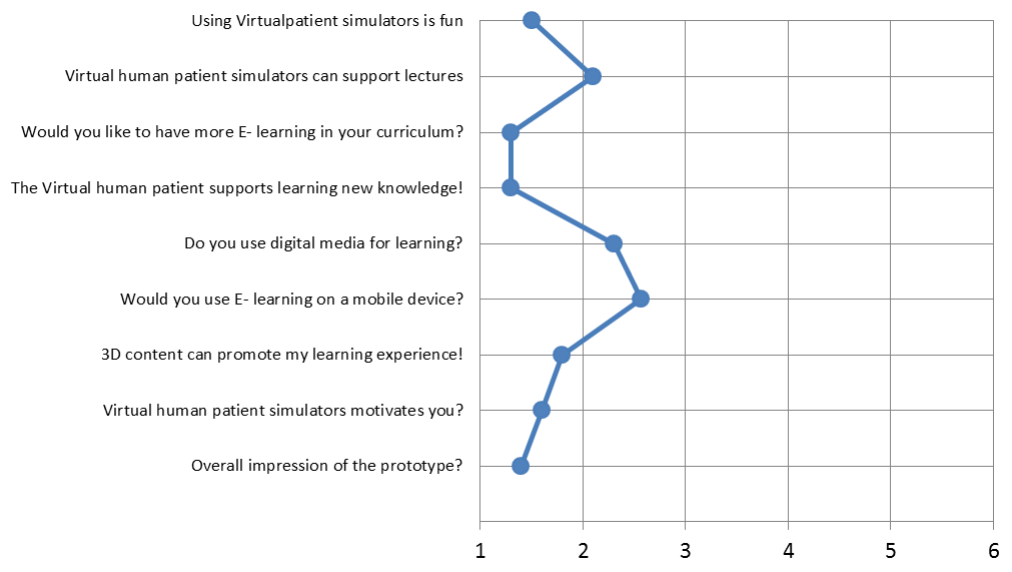
Acceptance, effectiveness, and applicability and preexisting computer affinity were determined using a Likert scale questionnaire where students had to mark one of 6 checkboxes for each question: 1: very reasonable, 2: mainly reasonable, 3: reasonable, 4: partially reasonable, 5: hardly reasonable, 6: not reasonable.

To test the hypothesis that simulator use has an impact on knowledge gain and students’ motivation, ALICE was validated in the form of an experimental study. The variable, simulator performance, was measured using the following parameters: correct order of the diagnostic pathway, correct or incorrect diagnosis, and correct therapy. ALICE stored the user behavior at the server level, logging students’ decisions. Students’ acceptance and their opinion about the effectiveness and applicability were determined by means of a questionnaire using a (forced choice) 6-point Likert scale (Figure 5). The variable “impact on future performance (predictive validity)” is an important parameter for simulator quality [14,15]. It was tested on several levels: (1) by comparison of pre- and postsimulator clinical reasoning files, (2) by comparison of simulator performance between Cases 1 and 3, and (3) by comparison of pre- and postsimulator multiple-choice questions. Student feedback was measured using a descriptive Likert scale. Evaluation was approved as a pilot project by the Educational Committee of the Medical Faculty at the University of Cologne. The Institutional Review Board was informed and there were no objections.

Evaluation of simulator acceptance and students’ opinions were analyzed using descriptive methods such as the Likert scale. Simulator performance was analyzed using the McNemar test. Performance in the pre- and postquestionnaire multiple-choice questions was analyzed using the Student *t* test. *P*˂.05 was considered significant. Data were analyzed using the SPSS software package version 20.

## Results

### Module Development

The content module contains the declarative and procedural knowledge of patients diagnosed with esophageal cancer. The diagnostic and therapeutic workflow of this disease is standardized. However, this disease requires an individual risk analysis as the chosen therapy depends on the tumor stage [16] and functional factors [17]. Although esophageal cancer is a rare disease in Europe, it is a common disease at our hospital. As a Center of Excellence for Surgery of the Esophagus and Stomach, we see more than 400 such patients and perform more than 160 esophagectomies annually. Because the topic of esophageal cancer was not part of the previous curriculum until that point, we were able to minimize influence of preexisting knowledge.

**Figure 6:**
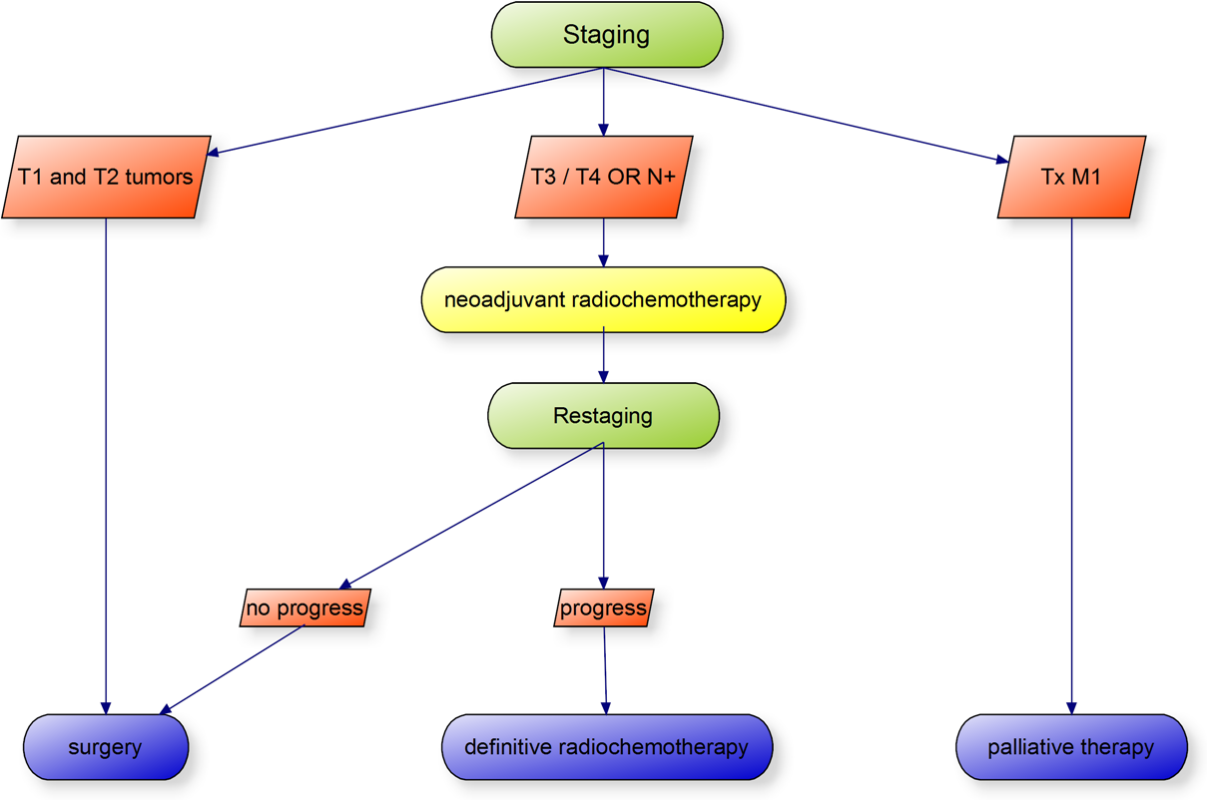
Therapeutic workflow based on German evidence-based guidelines. Parameters are tumor depth (T1 to T4), lymph node manifestation (N+ or N-), and metastasis (M1 or M0).

**Figure 7:**
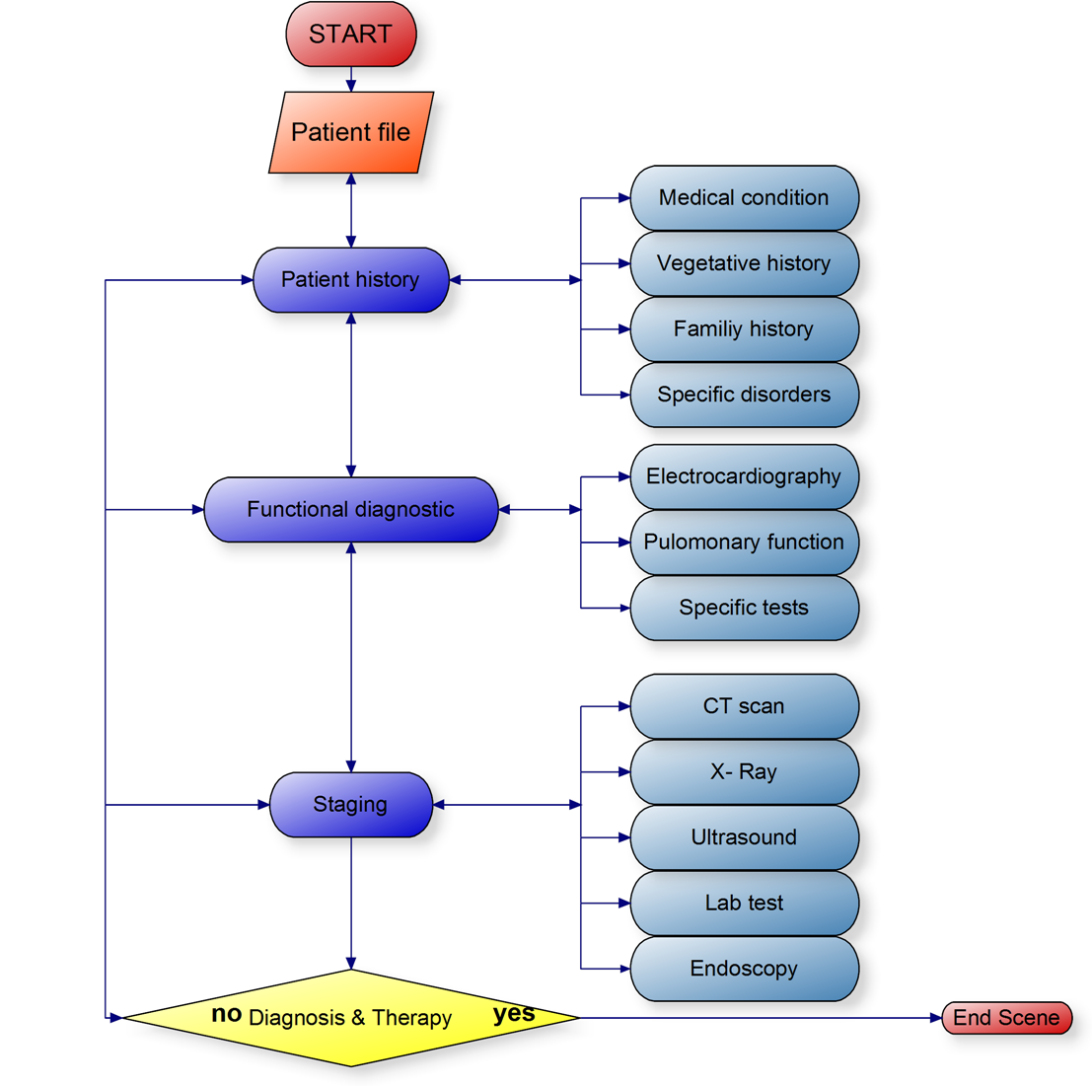
Template of an “optimal” diagnostic process (light and dark blue subitems can be freely chosen). Staging tests are again subdivided into more precise tests (eg, CT into “CT Abdomen,” “CT Thorax,” “PET CT”).

The esophageal cancer module was added to the ALICE framework and included 3 cases with different tumor stages and different therapeutic options (Table 1). More patients can easily be added when necessary. Declarative and procedural learning targets were based on the German medical examination regulations and served as a template for the procedural content. The correct diagnostic and treatment patterns were defined in process charts (Figures 6 and 7) by 2 different experienced clinical teachers and served as templates for the analysis of student performance. Clinical findings as well as corresponding images, videos, and tables from real patients were collected. Radiological images were interpreted by an experienced radiologist while gastroscopy videos were interpreted by an experienced endoscopist.

**Table 1:** Three cases with patients diagnosed with esophageal carcinoma.^a^

	Case 1	Case 2	Case 3
Diagnosis	Adenocarcinoma	Adenocarcinoma	Squamous cell carcinoma
Tumor stage	T3 Nx M0	T4 Nx M1	T2 Nx M0
Diagnostics	Standard	Plus PET computed tomography	Plus bronchoscopy
Therapy	Neoadjuvant	Palliative	Surgery
	Restaging		
	Surgery		

^a^The 3 cases differ in tumor stage and histology, and thus require a slightly different diagnostic pathway and therapeutical approach.

### Evaluation

A total of 62 students participated in the survey. Students’ acceptance and their opinion about the effectiveness and applicability were determined using a (forced choice) 6-point Likert scale. Six response categories were intentionally chosen because learning performance in Germany is traditionally measured on a 6-point scale and participating students were therefore familiar with a 6-level grading system. Likert scale assessment was averaged and is summarized in Figure 5. Students showed a high level of motivation when using the simulator as most of them had fun using it. Most of the students demand more e-learning in their medical curricula. These students showed a high acceptance of the simulator prototype and would frequently use a simulator of this kind. Most of the students use computers on a daily basis. The majority of the students agreed that ALICE taught new knowledge.

Predictive validity was tested by measuring the impact of simulator use on declarative knowledge. Ten multiple-choice questions that were asked before and after the simulation were correlated. In the prequestionnaire, the mean number of correct answers was 5 out of 10 (SD 1). In the postquestionnaire, the students achieved 7 out of 10 correct answers (SD 1). Hence, ALICE showed a significant impact on declarative knowledge gain (*P*<.01).

The impact of simulator use on future clinical decision making was tested by comparing clinical decision making before and after simulator use (Table 2). Students were asked to write down the most probable diagnosis and the suggested therapy. These answers were compared by the tutor and evaluated as “right” or “wrong.” In the prequestionnaire, 65% (40/62) of students chose the right diagnosis whereas after simulator use, 92% (57/62) of students chose the right diagnosis. In the prequestionnaire, only 32% (20/62) of students chose the right therapeutic concept, and in the postquestionnaire, 76% (47/62) of students prescribed the suitable therapy. As this improvement was significant, simulator use had a positive impact on clinical decision making.

**Table 2:** Detailed results from the pre- and postquestionnaire where the mean number of correct answers rose from 5/10 to 7/10 (*P*<.01).

Parameters	Correct answers, n		*P* value
	Presimulator	Postsimulator	
			
Number of multiple-choice questions (out of 10)	5 (SD 1)	7 (SD 1)	<.01
Students who made correct diagnosis (full text)	40	57	<.01
Students who choose right therapy (full text)	20	47	<.01
Students who made correct simulator diagnosis	48	52	Not significant
Students who chose correct simulator therapy	27	44	<.01
Students who chose correct pathway	23	21	Not significant

Reproduction of trained content was determined by comparing the simulator performance in Case 1 with Case 3 as these cases dealt with a similar diagnosis but different tumor stagings and different therapies. The correct pathway parameter was based on the flow diagram shown in Figure 6. Medically nonindicated tests as well as wrong sequence of performed tests resulted in a negative rating. Simulator use showed no positive impact on the correct pathway parameter. The diagnosis and therapy parameters were compared on students’ choices based on pull-down menus with single statements. The diagnosis parameter showed no significant improvement between the two simulator cases. However, students showed an improvement in performance for the right therapy parameter (*P*<.01).

## Discussion

### Principal Findings

This study describes the successful establishment of a simulator (ALICE) for learning decision making in oncological patients. Previous studies revealed that using virtual patient simulators can have a positive impact on learning success [18]. Immersive patient simulators enhance virtual patient simulators thanks to an immersive environment where the user becomes part of the simulation. There is evidence that immersion plays a fundamental role in virtual reality simulators, as identification with the avatar impacts motivation and improves learning success [7]. However, the degree of immersion is influenced by many factors and furthermore probably not all students are equally suited to this learning style [19].

For clinical teachers, information about validity and usability are important as new educational concepts compete against the established courses. Knowledge of the SOPs in diagnosis and therapy is essential when treating oncological patients [20]. Simulator design is based on the concept of Miller’s pyramid [11] (Figure 2) and was developed to support clinical education in the first three steps. Miller’s pyramid gives a vivid impression of the necessary steps in acquiring and assessing knowledge. While our simulator is designed to address levels one and two, level four (Action: Does) is assessed in vivo (eg, bedside teaching), and therefore cannot be addressed by our simulator. For level three (Shows how), the parameter on reproduction of trained content was used. However, performance on this level is usually assessed in vitro, for instance, in simulated patients in an Objective Structured Clinical Examination (OSCE) environment. It is questionable whether virtual patients can replace simulator patients in an OSCE environment. We cannot answer this question with this study as this would require a comparison of two randomly assigned groups (virtual versus OSCE patients).

However, clinical reasoning includes not only knowledge of SOPs but is also influenced by many other factors, as also summarized in Bloom’s learning taxonomy [12], which reveals the limitations of immersive virtual patient simulators in clinical education. In the cognitive domain, ALICE focuses on supporting foundation thinking (Figure 3) as training of critical thinking is hard to implement within a simulation.

Moreover, two other domains of Bloom’s taxonomy are not addressed by ALICE: in the affective and psychomotor domains, weighting clinical findings and evaluating different hypotheses, professional attitude, and behavior are key in clinical education and depend on the experience of the teacher. These skills are very difficult to impart using immersive virtual patient simulators. Therefore, the gold standard on clinical education is the small-group attendance learning [3] accompanied by a medical teacher [4,5].

Learning in small groups is impaired by the increasing workload of hospital doctors [21] and restrictive working time directives. Small-group learning is most effective when students have a similar knowledge level. Here, ALICE can potentially support clinical education as the simulator can prepare students for the attendance-based courses. Students can learn the reasoning of a practitioner at their own individual pace with repetition as required. Moreover, a performance-based assessment can test the students’ problem-solving skills [22] and ensure sufficient student preparation.

ALICE showed an impact on knowledge gain as students displayed a significant improvement in finding the right therapeutic concept after working with the simulator. This is also supported by other studies for different validity levels [18,23]. As these assessments are often focused on a specific part of procedural knowledge [24], we also measured knowledge gain in terms of declarative knowledge. The comparison of pre- and posttest performance revealed an increase in declarative knowledge.

ALICE uses a three-dimensional engine similar to computer games and enables an immersion that is known to have a positive impact on students’ motivation [25] and even learning performance [7]. In this study, students were enthusiastic and motivated while learning with ALICE. This was to be expected as this concept meets the expectations of Generation Y [26]. Nevertheless, this enthusiasm is a pitfall when it comes to analyzing student performance. Contrary to reality, students tended to deliberately select all available technical examination methods one after another as they were fascinated by the high number of images and videos. As students did not exclude nonmedically indicated tests, the comparison of simulator performance in terms of the correct pathway parameter between Cases 1 and 3 was not successful. Even when students were briefed before simulator use to select only the indicated tests, this behavior was detectable. Future simulator modification is necessary to motivate students to find the optimal way instead of clicking all available tests.

### Conclusion

ALICE is an appropriate educational tool for teaching procedural knowledge. It has a positive effect on knowledge gain and boosts student motivation. When used as required preparation in education, it can possibly lead to more efficient bedside teaching.

## Abbreviations

ALICE: Artificial Interface for Clinical Education
IPS: immersive patient simulator
OSCE: Objective Structured Clinical Examination
SOP: standard operating procedure
